# Changes of Patterns and Outcomes of Ocular and Facial Trauma Among Children in Jordan

**DOI:** 10.7759/cureus.16833

**Published:** 2021-08-02

**Authors:** Ahmed E Khatatbeh, Enas Othman, Ali M Alalawneh, Hiba A Khraisat, Samer Alawneh, Moiz Ahmed, Kiran Abbas

**Affiliations:** 1 Medicine, Queen Rania Al Abdullah Hospital for Children, Amman, JOR; 2 Dentistry, King Hussein Medical Center, Amman, JOR; 3 Ophthalmology, Queen Rania Al Abdullah Hospital for Children, Amman, JOR; 4 Ophthalmology, King Hussein Medical Center, Amman, JOR; 5 Medicine, Jinnah Postgraduate Medical Centre, Karachi, PAK; 6 Medicine and Surgery, Sindh Medical College, Karachi, PAK

**Keywords:** children, ocular trauma, patterns, facial trauma, ecchymosis

## Abstract

Introduction

The eye is the second most common organ affected by trauma after hands and feet. Eye trauma is a common cause of visual morbidity and may result in irreversible visual impairment and blindness. Ocular and facial trauma contribute to significant proportions of visual deficits among young children. This study aimed to explore the changes in trend including the pattern and outcomes of ocular and facial trauma among children between the last twenty years.

Methodology

A retrospective study was conducted between January 2020 and April 2021. The medical records of the patients who attended the Royal Medical Services (RMS) military hospital between January 1999 and December 2019 suffering from eye trauma which required hospitalization were enrolled in the study and reviewed regarding age, gender, mechanism of trauma, the severity of the trauma, eye structures involved, and visual outcome. The patients were divided into three groups based on the time of trauma: Group A for injuries in the period (January 1, 1999 to December 31, 2005), Group B for the period (January 1, 2006 to December 31, 2012), and Group C for the period (January 1, 2013 to December 31, 2019). The collected data was analyzed and compared to explore whether there is any change in the pattern and visual morbidity of eye injuries over time. The most frequent finding of eye injury was corneal wound in Groups A and B patients, while in Group C the most common ocular injury was ecchymosis or sub-conjunctival hemorrhage.

Results

Three-thousand one-hundred and thirty only patients (3130 eyes) aged between 2 and 14 years (mean 7.11 ± 3.13) were included in the study. The male to female ratio was 2:1. 1864 patients (56.6%) were at five years of age or younger. The most common place of injury in the three groups was on the street. This ratio decreased from 64.0% in Group A to 48.8% in Group C. Stone was the commonest etiology of injury in Group A (38.0%) while wood and fall were the commonest in Group B (28.5%) and Group C (37.1%) respectively. Open globe injuries constituted 67.0% of patients in Group A, 64.7% of patients in Group B, and 51.2% of patients in Group C. Normal or mild visual impairment was noted in patients of Group C (43.9%) as compared to the patients in Groups A (7.5%) and B (8.3%) at presentation. The final vision of normal or mild visual impairment was reported in 37.1%, 38.5%, and 77.5% of patients in Groups A, B, and C respectively.

Conclusion

The current study is a retrospective analysis of twenty years in Jordan and has comprehensively explored the trends and patient outcomes with respect to ocular and facial trauma among children. We revealed that over time, such injuries became less frequent and less serious than before with better patient outcomes. Furthermore, higher rates of closed globe injuries were reported in recent years. There was a dramatic increase in the rate of indoor injuries compared with outdoor ones which were mostly caused by falls with better initial and final visual outcomes. These injuries are preventable with the implementation of adequate safety measures which would significantly reduce the burden of visual impairment and cosmetic disfigurement among youngsters.

## Introduction

The eye is the second most common organ affected by trauma after hands and feet [1]. Eye trauma is a common cause of visual morbidity and may result in irreversible visual impairment and blindness [2-3]. In addition, eye trauma may have adverse economic and psychological impacts on individuals’ lives and their families [4]. Trauma is still the leading cause of unilateral blindness in children [5]. Up to 14% of pediatric patients suffer from severe visual impairment or blindness because of trauma. Children are more likely to be affected by trauma when compared to adults because of their limited skills and poor assessment of external hazards.

Trauma to the eye can be isolated or associated with other systemic injuries. It can also be divided according to the Birmingham Eye Trauma Terminology System (BETTS) into closed and open globe injuries [6-7]. Furthermore, chemical injuries are considered a separate entity of ocular injuries. Ocular trauma in developing countries like Jordan is difficult to be compared with that in developed countries because of the extreme difference in the lifestyle of individuals which may affect the rate, mechanism, and outcome of ocular trauma.

Due to the scarcity of data on the topic and outdated existing literature, we decided to conduct a retrospective analysis at our center in Jordan. This study was conducted to explore the patterns and outcomes associated with ocular and facial injuries in the last twenty years. The authors wanted to determine whether there were any significant changes in those parameters over time in Jordan.

## Materials and methods

This was a retrospective study conducted at the Royal Medical Services (RMS) hospital between January 2020 and April 2021. The cases of patients who attended RMS hospital between January 1999 to December 2019 suffering from eye and facial trauma that required hospitalization were studied. A non-probability convenience sampling technique was employed to recruit the cases into the study. Since the study was a retrospective analysis, ethical approval was not required; nevertheless, the topic was approved by the head of the department prior to the conduction of the study. All personal identifiers were removed and data was coded before the analysis was performed to make sure patient confidentiality was maintained throughout the study.

The medical records were reviewed for the patient's age, gender, and etiology, and type of trauma. The outcome of eye examination including best-corrected visual acuity (BCVA) using Snellen chart was reviewed at the time of presentation and after eight months as well. Data regarding the socio-economic feature of the family and the presence of an adult who was attending at the time of injury were recorded. The patients were divided into three equal groups according to the date of injury; Group A for injuries in the period (January 1999 to December 2005), Group B for the period (January 2006 to December 2012), and Group C for the period (January 2013 to December 2019). The results of the three groups were compared with each other to explore if there was any significant change in the rate, pattern, and outcome of ocular and facial trauma with the time period.

All data were entered into a predefined proforma, electronically and was later analyzed using Statistical Package for Social Sciences (SPSS). An independent t-test was used for the analysis of the obtained data. Descriptive analysis was used in the form of frequency, percentages, mean, and standard deviation. P-value of (< 0.05) and (<0.001) was considered to be significant and highly significant respectively.

## Results

A total of 3130 cases, aged between 2 and 14 years with a mean age of 7.11 ± 3.13 years were studied. The male to female ratio was 2:1. 1864 patients (56.6%) were at five years of age or younger. The demographic features of the patients are summarized in Table 1. The mean time of seeking medical care from the time of injury was significantly shorter (P-value < 0.001) in Group C as compared to Groups A and B.

**Table 1:** The Demographic Features of Children Suffering From Eye Injuries in All Groups n: Number of Patients

	Group A (n=1311) January 1, 1999 to December 31, 2005	Group B (n=1108) January 1, 2006 to December 31, 2012	Group C (n=711) January 1, 2013 to December 31, 2019	Total (n=3130)
2 to ≤5 years	717 (54.7%)	654 (59.0%)	493 (69.3%)	1864 (56.6%)
>5 to ≤10 years	484 (36.9%)	372 (33.6%)	175 (24.7%)	1031 (32.9%)
>10 years	110 (8.4%)	82 (7.4%)	43(6.0%)	235 (7.5%)
Total	1311 (100%)	1108 (100%)	711 (100%)	3130 (100%)
Males	874 (66.7)	744 (67.1%)	472(66.4%)	2090 (66.8%)
Females	437(33.3%)	364 (32.9%)	239 (33.6%)	1040 (33.2%)
Mean-time of seeking medical care (hours)	56.2±12.3	47.7±9.3	26.1±11.2	46.4±19.2

None of the parents in 73.3% of patients in Group A finished the higher secondary school education compared to 59.1% and 49.1% of patients in Groups B and C respectively. On the other hand, a higher level of parent’s education was seen in 26.7% and 50.9% of patients in Groups A and C respectively. The family income was assessed in the three groups and compared with the mean level of income at that particular time reported by the Jordanian National committee of statistics; average or poor income was slightly higher in Groups B and C than in Group A, which is not statistically significant (p-value >0.564). The results of family income and parent’s level of education are summarized in Table 2.

**Table 2:** The Results of Family Income and Parent’s Level of Education in Groups A, B, and C. n: Number of Patients

		Group A (n=1311) January 1, 1999 to December 31, 2005	Group B (n=1108) January 1, 2006 to December 31, 2012	Group C (n=711) January 1, 2013 to December 31, 2019	Total (n=3130)
Parent’s level of education	Illiterate	131 (9.9%)	67 (6.0%)	22 (3.1%)	220 (7.0%)
	Up to Secondary school	832 (63.4%)	588 (53.1%)	327 (46.0%)	1747 (55.8%)
	College or University	335 (25.6%)	440 (39.7%%)	350 (49.2%)	1125 (35.9%)
	PhD	13 (1.1%)	13 (1.2%)	12 (1.7%)	38 (1.3%)
Total		1311 (100%)	1108 (100%)	711 (100%)	3130 (100%)
Family income	Poor	402 (30.7%)	350 (31.6%)	249 (35.0%)	1001 (32.0%)
	Average	726 (55.3%)	625 (56.4%)	403 (56.7%)	1754 (56.0%)
	High	183 (14.0%)	133 (12.0%)	59 (8.3%)	375 (12.0%)
Total		1311 (100%)	1108 (100%)	711 (100%)	3130 (100%)

The street is the most common place of injury in the three groups. However, this ratio decreased from 64.0% in Group A to 48.8% in Group C. Indoor injuries increased from 28% in Group A to 44.3% in Group C (p-value <0.021). On the other hand, there was no statistical difference between the three groups in the rate of injuries that happened at school (P-value >0.124). The results are summarized in Table 3. Adults were not present at the place of injury during its occurrence in 72.7% of the total cases. This percentage was highest in Group A (79.1%) and lowest in Group C (59.8%). The absence of supervising adults was significantly associated with a low level of parent’s education.

**Table 3:** The Place of Injury Distribution in All Groups. n: Number of Patients

Place of injury	Group A (n=1311) January 1, 1999 to December 31, 2005	Group B (n=1108) January 1, 2006 to December 31, 2012	Group C (n=711) January 1, 2013 to December 31, 2019	Total (n=3130)
Home	367 (28.0%)	355 (32.0%)	315 (44.3%)	1037 (33.1%)
Street	839 (64.0%)	676 (61.0%)	340 (48.8%)	1855 (59.3%)
School	105 (8.0%)	77 (7.0%)	56 (7.9%)	238 (7.6%)
Total	1311 (100%)	1108 (100%)	711 (100%)	3130 (100%)

The time of injury during the year was highest in Summer with no significant difference between the groups. Stone was the commonest etiology of injury in Group A (38.0%) while wood and fall were most common in Group B (28.5%) and C (37.1%) respectively. The etiologies of injury are summarized in Table 4.

**Table 4:** Etiology of Eye Injuries in Groups A, B, and C. n: Number of Patients

Etiology of injury	Group A (n=1311) January 1, 1999 to December 31, 2005	Group B (n=1108) January 1, 2006 to December 31, 2012	Group C (n=711) January 1, 2013 to December 31, 2019	Total (n=3130)
Wood	420 (32.0%)	316 (28.5%)	141 (19.8%)	877 (28.0%)
Fall	211 (16.1%)	245 (22.1%)	264 (37.1%)	720 (23.0%)
Toy Guns	93 (7.1%)	101 (9.1%)	121 (17.0%)	315 (10.1%)
Stone	249 (38.0%)	188 (17.0%)	63 (8.9%)	500 (16.0%)
Metal wire	182 (13.9%)	135 (12.2%)	50 (7.0%)	367 (11.7%)
Knife	52 (4.0%)	45 (4.1%)	21 (3.0%)	118 (3.7%)
Glass	40 (3.1%)	26 (2.3%)	20 (2.8%)	86 (2.7%)
Nail	26 (2.0%)	25 (2.2%)	14 (2.0%)	65 (2.1%)
Chemical	25 (1.9%)	23 (2.1%)	13 (1.8%)	61 (2.0%)
Others	13 (1.0%)	4 (0.4%)	4 (0.6%)	21 (0.7%)
Total	1311 (100%)	1108 (100%)	711 (100%)	3130 (100%)

Open globe injuries constitute 62%.6 of the total cases and constitute the most common mode of injury in all groups. However, its frequency was slightly lower at a rate of 64.7% in Group B compared with 67.0% in Group A and significantly lower in Group C at a rate of 51.2% (P-value <0.001). There was no significant difference regarding chemical injuries among the three groups (P-value >0.119. In Group A, 665 patients (75.7%) of open globe injures did not finish the high secondary school education while in closed globe injury, only 167 patients (40.1%) did not finish the high secondary school education**.** On the other hand, in Group C patients this rate was 72.5% and 19.5% in open and closed globe injuries respectively. The types of ocular injuries and their rates are summarized in Table 5.

**Table 5:** Types of Ocular Injuries in Groups A, B, and C. n: Number of Patients

	Group A (n=1311) January 1, 1999 to December 31, 2005	Group B (n=1108) January 1, 2006 to December 31, 2012	Group C (n=711) January 1, 2013 to December 31, 2019	Total (n=3130)
Open	878 (67.0%)	717 (64.7%)	364 (51.2%)	1959 (62.6%)
Closed	408 (31.1%)	368 (33.2%)	334 (47.0%)	1110 (35.5%)
Chemical	25 (1.9%)	23 (2.1%)	13 (1.8%)	61 (1.9%)
Total	1311 (100%)	1108 (100%)	711 (100%)	3130 (100%)

The most frequent finding of eye injury was corneal wound in Groups A and B patients while in Group C, the most common ocular injury was ecchymosis or sub-conjunctival hemorrhage followed by hyphema. Corneal abrasion was the most common finding in chemical types of injuries while corneal wound and hyphema were the most common finding in open and closed globe injuries, respectively. Table 6 represents the spectrum of ocular findings among patients with ocular trauma.

**Table 6:** Ocular Findings Among Patients With Ocular Trauma. n: Number of Patients

Outcome of injury	Group A (n=1311) January 1, 1999 to December 31, 2005	Group B (n=1108) January 1, 2006 to December 31, 2012	Group C (n=711) January 1, 2013 to December 31, 2019	Total (n=3130)
Lid and or conjunctival laceration	111 (8.5%)	98 (8.8%)	92 (12.9%)	301 (9.6%)
Ecchymosis or sub-conjunctival hemorrhage	256 (19.5%)	232 (20.1%)	227 (31.9%)	715 (22.8%)
Corneal abrasions	84 (6.4%)	74 (6.7%)	86 (12.1%)	244 (7.8%)
Corneal foreign body	105 (8.0%)	85 (7.8%)	94 (13.2%)	284 (9.1%)
Full-thickness corneal wound	425 (32.4%)	313 (28.2%)	135 (19.0%)	873 (27.9%)
Corneal scleral wound	337 (25.7%)	281 (25.4%)	131 (18.4%)	749 (23.9%)
Scleral wound	51 (3.9%)	37 (3.3%)	12 (1.7%)	100 (14.1%)
Hyphema	237 (18.0%)	197 (17.8%)	171 (24.1%)	605 (19.3%)
Iridodialysis or sphincter tear	59 (4.5%)	46 (4.2%)	19 (2.7%)	124 (4.0%)
Cataract	384 (29.3%)	309 (27.9%)	160 (22.5%)	853 (27.3%)
Lens subluxation	89 (6.8%)	79 (7.1%)	13 (1.8%)	181 (5.9%)
Vitreous hemorrhage	97 (7.4%)	77 (6.9%)	31 (4.4%)	205 (6.5%)
Intra-ocular foreign body	32 (2.4%)	26 (2.3%)	6 (0.8%)	64 (2.0%)
Retinal breaks	24 (1.8%)	16 (1.4%)	5 (0.7%)	45 (1.4%)
Retinal detachment	16 (1.2%)	12 (1.1%	2 (0.3%)	30 (1.0%)
Endophthalmitis	4 (0.3%)	3 (0.3%)	1 (0.1%)	8 (0.3%)
Phthisis	3 (0.2%)	2 (0.1%)	0 (0.0%)	5 (0.2%)
Bone fracture	15 (1.1%)	13 (1.2%	7 (1.0%)	35 (1.1%)
Tooth avulsion or fracture	6 (0.5%)	5 (0.5%)	3 (0.4%)	14 (0.5%)
Tongue laceration	5 (0.4%)	4 (0.4%)	2 (0.3%)	11 (0.4%)

There was significant improvement of final BCVA compared with vision at presentation in all the groups (P-value <0.01). In addition, the mean initial and final visual acuity in Groups A and B were comparable but slightly better in Group B with no statistical difference (P-value >0.01). On the other hand, Group C patients showed highly significantly better initial visual and final BCVA when compared with Groups B and C (P-value <0.001). More than 90% of cases of Groups A and B had moderate or severe visual impairment at presentation while severe final visual impairment was found in nearly 60% of those cases. In Group C patients, only 54.2% and 22.5% had severe visual impairment at presentation and eight months after trauma, respectively. The results of visual acuity examination for all patients are summarized in Table 7.

**Table 7:** BCVA Among Eyes With Ocular Trauma in All Groups. n: Number of Patients, BCVA: Best-corrected visual acuity, PL: Perception of light, NPL: No perception of light

Mean BCVA	Group A (n=1311) January 1, 1999 to December 31, 2005		Group B (n=1108) January 1, 2006 to December 31, 2012		Group C (n=711) January 1, 2013 to December 31, 2019	
	Initial	Final	Initial	Final	Initial	Final
0.6-1.0	20 (1.5%)	188 (14.3%)	21 (1.9%)	166 (15.0%)	111 (15.6%)	221 (31.1%)
<0.6-≤0.3	79 (6.0%)	299 (22.8%)	71 (6.4%)	260 (23.5%)	201 (28.3%)	330 (46.4%)
<0.3-≤0.1	255 (19.5%)	313 (23.9%)	224 (20.2%)	276 (24.9%)	132 (18.6%)	98 (13.8%)
<0.1-PL	957 (73%)	506 (38.6%	791 (71.4%)	405 (36.6%)	267 (37.6%)	62 (8.7%)
NPL	0 (0%)	5 (0.4%)	1 (0.1%)	1 (0.1%)	0 (0.0%)	0 (0.0%)

Surgical intervention was performed in 984 patients (75.1%), 812 (73.3%), and 452 (63.6%) in Groups A, B, and C respectively. The most common operation performed was rupture globe repair followed by cataract surgery and pars plana vitrectomy.

## Discussion

Ocular trauma is a known cause of unilateral visual morbidity in children. It was found to be the leading cause of non-congenital unilateral blindness in people under twenty years of age in the United States. Children are more vulnerable to ocular trauma as compared to adults because of their poor ability to estimate the external hazards and to assess the associated risks [8]. In addition, their motor skills are not well developed to deal with such hazards [6,9]. Many studies were conducted to evaluate ocular trauma in children regarding epidemiology, etiology, mechanism, and visual outcome [10-11]. This retrospective study focused on those parameters over years to explore whether there were any alterations in those patterns with time.

Despite the increase in the number of the Jordanian population over the last two decades, there was a significant reduction in the number of eye injuries; the number of patients exposed to ocular injury dropped from 1113 patients (between January 1, 1999 to December 31, 2005) to 711 patients (between January 1, 2013 to December 31, 2019). In this study, children at five years of age or below were the most frequent individuals exposed to ocular injuries at a rate of 56.6%. The rate was higher in Group C when compared with Groups A and B. Comparable results were obtained by El-Sebaity et al. and Mayek et al. [5,12]. Children at this age compared to older individuals have immature mental and physical skills which make them unable to assess and face surrounding hazards. In contrast to those results, Al-Bdour found the highest incidence of injuries to be among children of six to ten years of age [13]. In addition, Bućan et al reported the highest incidence of injuries among children aged ten to fourteen years [8]. In the present study, males were twice affected as females with no significant variation between the three groups. Male predominance is related to the type of activity performed by them, which is quietly different from that of females. In addition, males are allowed to spend more time in the street which results in more exposure to surrounding dangers. A higher male ratio was found in most of the other studies but with variable percentages; it ranged between 2:1 to 4.5:1 [8,14-15].

The mean time for seeking medical care obviously declined over time: it was 56.2 hours in Group A and became 47.7 hours and 26.1 hours in Groups B and C, respectively. This significant decrease is probably related to improvement in transportation, better availability of health care units, and the great progress in the level of education among the Jordanian society [16,17-18]. The improvement of the education level among the Jordanian society was noted among parents in our sample as well; only 26.7% of parents in Group A were able to get at least a college or university degree compared with 40.9% and 50.9 % in Groups B and C respectively. Low parents’ level of education was found to be associated with higher incidence and more severity of ocular injuries and worse visual prognosis [19-20]. Although the education level of the parents of the patients in Group C was higher than those in Groups A and B, according to the general Jordanian population, this proportion is still crucially low.** **This emphasizes that the education level of the parents may influence the rate and outcome of ocular injuries. In contrast to that, there was an increase in the frequency of families with poor financial income among the study sample. In the latest years, 35.0% of Group C patients had slightly lower income compared to Groups A (30.7%) and B (31.6%) patients, respectively. Despite this, the incidence of ocular injuries was lower in Group C. This suggests that other factors played a role and influenced the outcome of trauma in Group C and this change is most likely to reflect the worsening of the financial status of Jordanian individuals which was reported by the Jordanian Department of Statistics [21]. In all groups, most of the patients came from families with low or average incomes. Many studies showed a strong relationship between the socio-economic level and the rates of ocular injuries [22-23].

Most ocular injuries in the three groups occurred on the street. However, the incidence decreased from 64.0% in Group A to 48.8% in Group C. This was combined with a significant increase in the frequency of ocular injuries which occurred at home. There were no changes in the rate of injuries that occurred at school. A lot of reports showed a recent increase in the time spent indoors than that spent in the street by children [24]. This change in the lifestyle of children may explain the significant reduction in the rates of outdoor ocular injuries and the increase of indoor ocular injuries. In addition to that, researchers reported an alteration in the sort of games played by children at home where electronic games which do not require any physical activity substituted the ordinary games with their associated aggressive activities [25-26]. This all explains the significant decrease in the rates and severity of ocular injuries like open globe injuries with an increase in the rates of closed globe injuries among patients in Group C. Playing electronic games probably contributed to the reduction in the incidence of ocular injuries among children above five years of age which resulted in an increase of the rates of ocular injuries among younger children who are unlikely playing those games. Stone was the most common etiology in Group A (38%) while fall was most commonly seen in Group C (37.1%). That’s because injury by stone is most likely to occur in the street which constitutes 64% of cases while falls can occur in either outdoor and indoor injuries.

This study also showed an obvious change in the types of ocular injuries; open globe injuries constitute two-thirds of injuries in Group A, and this rate dropped to one-half in patients of Group C. This change may be attributed to many factors such as the change in place of injury being more frequent at home which will result in lower exposure to severe injuries, the change in the type of patient’s activities which became less aggressive, and the change in the etiology of trauma from stone in Group A to fall in Group C. These all resulted in lower chance to get severe and serious ocular injuries. There was no change in the rates of chemical injuries in the three groups since accidental exposure is likely to be the culprit.

The most frequent finding of eye injury was corneal wound in Groups A and B patients while in Group C the most common ocular injury was Ecchymosis or sub-conjunctival hemorrhage followed by hyphema. This is because higher rates of open globe injuries were encountered in Groups A and B compared with the lower impact of trauma in Groups C. In the majority of ocular injuries absence of supervising adult at the time of injury was noted which was prominent in Group A patients (79%.1). This ratio was less in Group C, which may be attributed to the improved level of education in society. Despite this, lack of supervision for children particularly young ones seems to be an important risk for incidence and severity of ocular trauma. Therefore, paying attention to children may greatly prevent or minimize the incidence and severity of ocular trauma.

As seen with other studies, there was a significant improvement of final BCVA when compared to the initial BCVA [27-28]. Open globe injuries, poor initial BCVA, and late attendance were significantly associated with poor final visual outcomes (P-value <0.05%). This explains the great improvement in vision noticed in Group C when compared to Groups A and B. In addition, this study uniquely showed that the initial visual acuity was much better in Group C than those in Groups A and B; normal or mild visual impairment was noted in 43.9% of patients in Group C while only 7.5% and 8.3% of patients in Groups A and B, respectively had mild or moderate visual impairment. Regarding their final vision, normal or mild visual impairment was reported in 37.1%, 38.5%, and 77.5% of patients in Groups A, B, and C respectively.

Finally, surgical intervention was less commonly performed in Group C (63.6%) compared to Groups A (75.1%) and B (73.3%) because closed globe injuries were more commonly encountered in this group than open injuries which required immediate surgical intervention. The results of this study were supported by the previous studies in which it was concluded that ocular trauma can be prevented by improving the socioeconomic level of the entire population.** **Over the last twenty-one years, this study was able to detect a significant change in the incidence, mechanism, place, type, and visual outcome. Ocular injuries became less frequent than before with earlier attendance for medical care. The injuries became less serious. Higher rates of closed globe injuries were recorded which mostly occurred at home and were caused by falls. A better visual prognosis was noted among those patients.

## Conclusions

The current study is a retrospective analysis of twenty years in Jordan and has comprehensively explored the trends and patient outcomes with respect to ocular and facial trauma among children. We revealed that over time, such injuries became less frequent and less serious than before with better patient outcomes. Furthermore, higher rates of closed globe injuries were reported in recent years.

There was a dramatic increase in the rate of indoor injuries compared with outdoor ones which were mostly caused by falls with better initial and final visual outcomes. These injuries are preventable with the implementation of adequate safety measures which would significantly reduce the burden of visual impairment and cosmetic disfigurement among youngsters.
